# A Case Report on Breast Cancer Following Mantle Radiation for Hodgkin Lymphoma: Screening and Management

**DOI:** 10.7759/cureus.62584

**Published:** 2024-06-18

**Authors:** Yasmeen Mansour, Akintunde Akinleye

**Affiliations:** 1 Medicine, Sovah Health, Danville, USA; 2 Sovah Cancer Center, Sovah Health, Danville, USA

**Keywords:** breast cancer screening, screening mammogram, hodgkin lymphoma, hodgkin disease (hd), secondary cancer, screening, breast cancer, mantle radiation, mantle field radiation

## Abstract

Hodgkin lymphoma survivors who received mantle radiation are at risk of developing secondary malignant neoplasms. There is no established recommended screening guideline for this population. We discuss the case of a patient with a history of Hodgkin lymphoma status post-mantle field radiation, thyroid cancer status post-thyroidectomy, and now breast cancer following mantle radiation. The risk of adverse effects from mantle field radiation is well documented and includes secondary cancers of the thyroid, breast, lung, and cardiovascular disease. Advances in technology have led to an international paradigm shift in the management of Hodgkin lymphoma to reduce the diameter and dose of radiation based on the patient’s anatomy. However, there is no consensus regarding the optimal frequency or modality of breast cancer screening in patients with Hodgkin lymphoma status post-mantle radiation who are now in remission. We discuss screening methods for this population, which has a high risk of developing breast cancer, and emphasize the need for personalized medicine.

## Introduction

Hodgkin lymphoma was treated with mantle field radiation from the 1960s to as recent as the 1990s. Patients who received mantle radiation (radiotherapy to the neck, chest, and axilla) when it was more common and survived could be as young as 25 years of age now and over 70 years of age. The literature has established that this population has an above-average risk for developing secondary cancers, including breast, thyroid, and lung cancers, and cardiovascular disease [1]. The international community is exploring ways to minimize the acute and chronic adverse effects of both radiation and chemotherapy management of Hodgkin lymphoma [2-5]. Modern advances in technology have allowed for a paradigm shift in personalized radiation that minimizes the radiation dose to the surrounding anatomy.

As the field of medicine advances toward personalized disease management, it also holds the potential for personalized screening. The American College of Radiology (ACR) recommends that women with a history of mantle radiation, in addition to annual screening mammography, should receive supplemental screening contrast-enhanced breast MRI [6]. While the recommendation for women of average risk is to start screening at the age of 40, women with a history of mantle radiation are considered high risk and should begin screening earlier. By the age of 30, all women should be evaluated for breast cancer risk to stratify those at high risk so that they can start supplemental screening. The medical community has not reached a consensus, however, on the screening frequency and modality of patients who received mantle radiation.

## Case presentation

An adult female with a past medical history of essential hypertension, hyperlipidemia, depression, Hodgkin disease status post-splenectomy and mantle radiation, in remission, and thyroid cancer status post-total thyroidectomy, in remission, presented to her primary care physician for a painful left breast lump. The patient had not received annual screening mammograms. The mammogram found a 2 cm density in the right upper outer quadrant and a 3 cm density in the left upper outer quadrant of the left breast, which correlates to the palpable lump. The subsequent right breast ultrasound found a 14 x 12 x 8 mm lesion at 10 o'clock, 13.5 cm from the nipple. The left breast ultrasound found a 2.4 x 2.5 x 2.1 cm lesion at 1 o'clock, 10 cm from the nipple. BI-RADS (breast imaging-reporting and data system) was 5, and a biopsy was recommended.

An ultrasound-guided core needle biopsy of the right breast lesion found high-grade infiltrating ductal carcinoma, estrogen receptor (ER) negative, progesterone receptor (PR) negative, and human epidermal growth factor receptor 2 (HER2) negative. An ultrasound-guided core needle biopsy of the left breast lesion found ER-, PR-, HER2+ Grade 3 invasive ductal carcinoma with micropapillary features. A post-biopsy right breast mammogram found that the HydroMARK clip was positioned appropriately for the right upper outer quadrant spiculated nodule.

**Figure 1:**
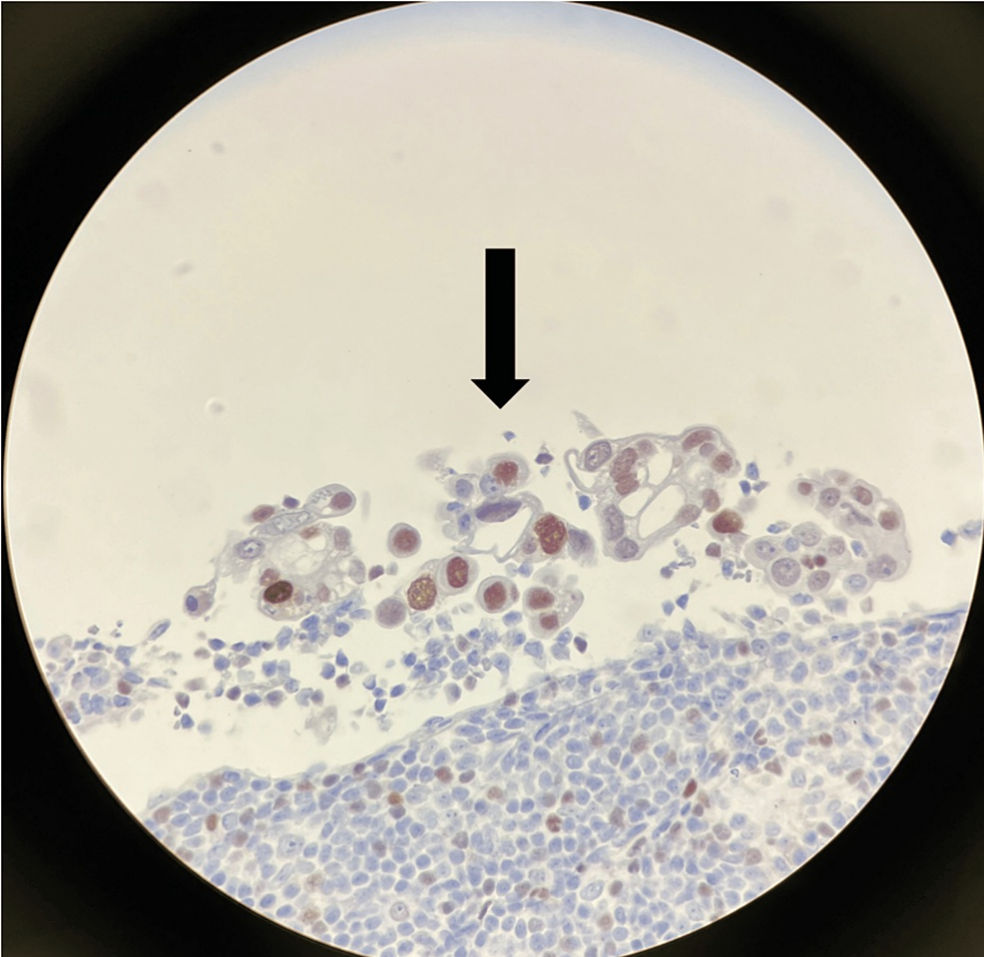
GATA 3 stain positive in adenocarcinoma, which is consistent with metastatic carcinoma of the breast, as indicated by the arrow.

**Figure 2:**
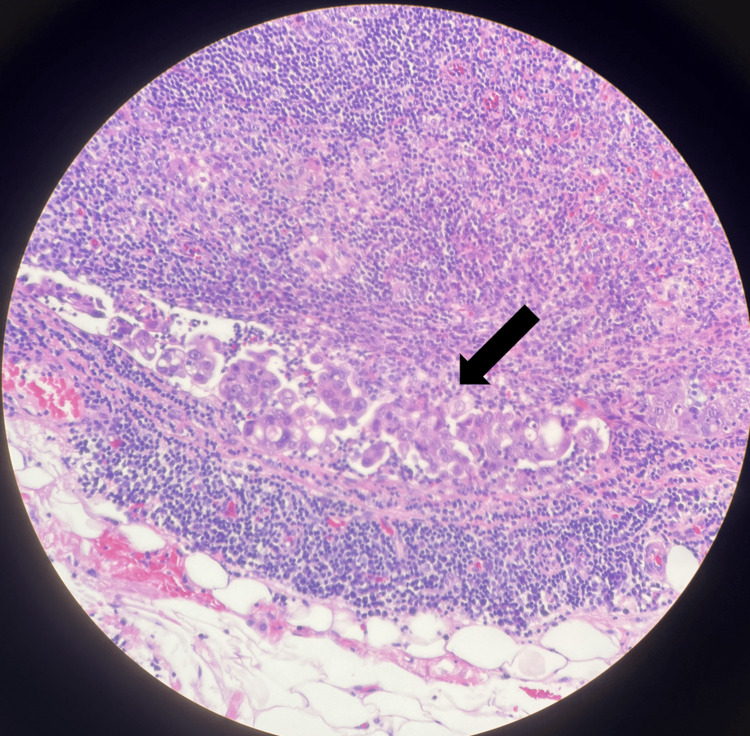
Hematoxylin and eosin (H&E) stain of metastatic carcinoma in the R10 lymph node, as indicated by the arrow.

**Figure 3:**
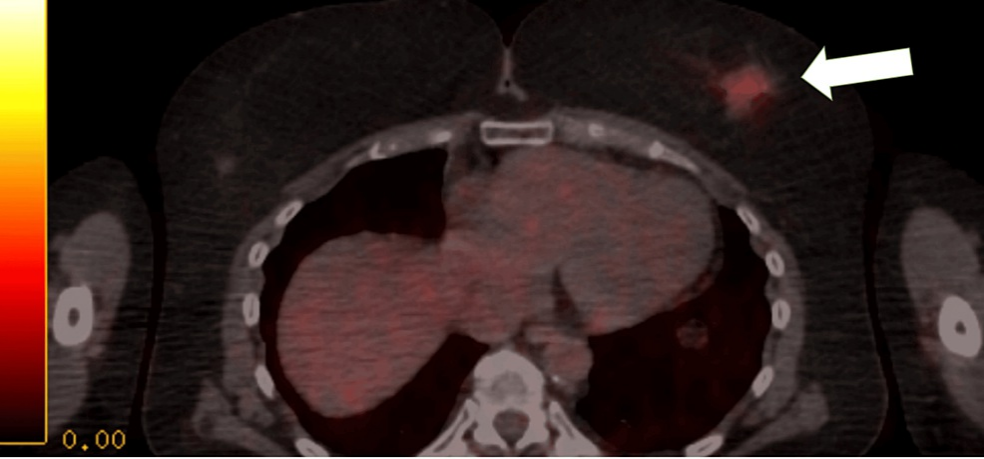
The arrow indicates the left upper outer breast mass.

**Figure 4:**
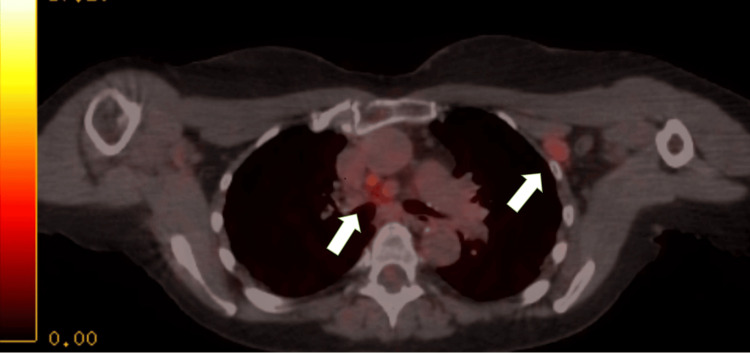
The left arrow indicates a subpectoral lymph node, and the right arrow indicates a mediastinal lymph node.

General surgery evaluated the patient and ordered a positron emission tomography-computed tomography (PET-CT) scan, which found a spiculated left upper outer breast mass measuring 2.7 cm, an enlarged 1.7 cm left subpectoral lymph node with a standard uptake value (SUV) max of 5.7, and multiple right hilar and mediastinal lymph nodes, as seen in Figures 1-4. A 1.3 cm precarinal lymph node was also identified with an SUV max of 6.2. The patient was discussed at the tumor board and was recommended for mediastinoscopy with biopsy and pathological evaluation of mediastinal lymph nodes with a subsequent image-guided biopsy of the left subpectoral lymph node.

**Figure 5:**
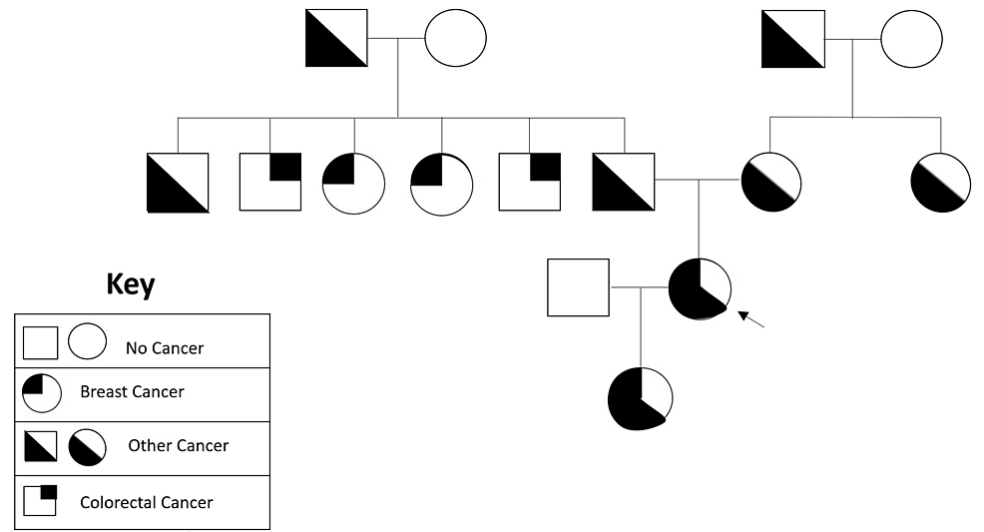
The pedigree of family cancer history. The arrow indicates the presence of breast cancer in the female patient, along with other cancers such as thyroid and Hodgkin lymphoma.

The patient was referred to and seen in the oncology clinic. The level of cancer antigen 15-3 elevated at 31.5 U/mL. The patient’s family cancer history pedigree is extensive, as seen in Figure 5. Despite an extensive family history, the genetic testing found her only to be heterozygous for the p.M1113T variant in the *MSH3* gene, which is of unknown significance. She was otherwise negative for the following 36 genes: *APC*, *ATM*, *AXIN2*, *BARD1*, *BMPR1A*, *BRCA1*, *BRCA2*, *BRIP1*, *CDH1*, *CDK4*, *CDKN2A*, *CHEK2*, *DICER1*, *MLH1*, *MSH2*, *MSH3*, *MSH6*, *MUTYH*, *NBN*, *NF1*, *NTHL1*, *PALB2*, *PMS2*, *PTEN*, *RAD51C*, *RAD51D*, *RECQL*, *SMAD4*, *SMARCA4*, *STK11*, and *TP53* (sequencing and deletion/duplication); *HOXB13*, *POLD1*, and *POLE* (sequencing only); and *EPCAM* and *GREM1* (deletion/duplication only). The patient confirmed that the previous mantle radiation did include the site of origin of her new breast cancer.

A mediastinoscopy with a biopsy of the mediastinal lymph nodes was performed. The pathology found ER-, PR-, HER2+ carcinoma in the mediastinal lymph nodes consistent with Stage IV metastatic disease. A Port-a-cath was inserted by general surgery. The patient was started on neoadjuvant chemotherapy plus immunotherapy to include carboplatin, docetaxel, trastuzumab, and pertuzumab every three weeks for six treatments. The plan is that the patient will subsequently undergo left axillary lymph node dissection and left mastectomy, followed by adjuvant immunotherapy.

## Discussion

There has been an international paradigm shift in how Hodgkin lymphoma is treated, from mantle field radiation therapy to smaller diameters and intensities of radiation so that there is minimal radiation to surrounding organs. At Rigshospitalet in Copenhagen, Denmark, their sample consisted of 10 patients who were diagnosed with Stage I-II Hodgkin lymphoma at 18 years of age or younger between 2005 and 2010 and who received radiation therapy [2]. FDG PET-CT was performed prior to chemotherapy, and CT with contrast was performed after chemotherapy. Various radiation therapy fields were simulated for each patient based on imaging. The differences in excess risk estimates and mean doses to organs were assessed via repeated measures analysis of variance (rANOVA). They found that the estimated radiation dose to the thyroid, breast, lungs, and heart could be reduced even in the case of mediastinal disease and, therefore, recommend that radiotherapy be considered for pediatric and adolescent patients.

In line with the international effort to minimize the adverse effects of radiation by reducing the radiation field and practicing personalized medicine, a single-center retrospective study in Japan evaluated patients between 2003 and 2016 who received dose-attenuated chemotherapy of doxorubicin, bleomycin, vinblastine, and dacarbazine (ABVD), which is the standard regimen [4]. They found that comorbidities and prognostic factors should be assessed when determining the degree of attenuation. Patients who did not have poor prognostic factors and who presented at an early stage of disease had a five-year progression-free survival of 92.9% (95% confidence interval, 59.1%-99%) and an overall survival of 100%.

Hodgson et al. propose individualized predictive risk estimates for radiation-associated second cancer risk in Hodgkin lymphoma patients based on radiation dose and field received using a novel model of carcinogenesis [5]. Their sample consisted of 37 patients at Princess Margaret Hospital in Canada who had Stage I-III Hodgkin lymphoma. They found that 35 Gy involved-field radiation therapy predicted a 20-year excess relative risk (ERR) reduction of breast cancer by 63% when compared to 35 Gy mantle radiation therapy. Meanwhile, low-dose (20 Gy) involved-field radiation therapy had an associated 77% decrease in ERR. Incorporating patient-specific differences in tissue doses further found an 11-fold variation in individual estimates of breast cancer ERR. Predictive estimates could further personalize and optimize radiation therapy of Hodgkin lymphoma to reduce the risk of secondary cancer.

While the international literature has established that mantle radiation is no longer the mainstay of treatment for Hodgkin lymphoma, there are diverse opinions regarding breast cancer screening in the United States among prominent organizations such as the United States Preventative Service Task Force (USPSTF), ACR, and the American College of Obstetricians and Gynecologists. The goal has been to improve mortality and reduce overdiagnosis, which refers to the diagnosis of breast cancer that otherwise would not have interfered with the patient’s quality of life or lifespan if left undetected and overtreatment. The Centers for Disease Control and Prevention (CDC) lists the USPSTF recommendations on their website but also includes a table of recommendations from various organizations [7].

The USPSTF gives a Grade B recommendation that women 50-74 years of age should receive biennial screening mammography [8]. Their recommendations for women less than 50 are essentially based on patient autonomy and personalized risk assessment. The USPSTF emphasizes that there is still a dearth of evidence available to determine if women 75 years of age or older should receive screening mammography or if women with dense breasts would benefit from screening with MRI, breast ultrasound, or digital breast tomosynthesis if they have a negative screening mammogram.

The CDC references a study conducted by Travis et al., which evaluated 3817 women who received treatment for Hodgkin lymphoma when they were 30 years of age or younger between 1965 and 1994 who survived at least one year from tumor registries in Denmark, Sweden, Finland, Iowa, and Ontario [9]. Through a nested case-control study, they calculated the cumulative absolute breast cancer risk for patients, which they found varied based on age at the end of the follow-up period, type of therapy received, and time since diagnosis. They suggested that their projections can be used to assist Hodgkin lymphoma patients who received treatment in the past when determining breast cancer screening regimens. However, this study population did not include women in the United States.

Clearly, there is insufficient evidence to determine when to initiate breast cancer screening, the frequency, and modality of screening for Hodgkin lymphoma survivors who received mantle radiation from the 1960s to 1990s in the United States. Early counseling and discussion with Hodgkin lymphoma patients status post-mantle radiation should emphasize they have an above-average risk of developing breast cancer, and their recommended screening regimen should be developed through shared decision-making while respecting their autonomy.

Summary of the case

This adult female developed thyroid cancer requiring thyroidectomy and now triple-negative breast cancer in the right breast and high-grade left breast infiltrating ductal carcinoma ER/PR- and HER+ from mantle radiation she received for Hodgkin disease. Unfortunately, this patient had not received screening mammography despite having an above-average risk of developing breast cancer. She felt a painful lump in her breast and subsequently sought care. An imaging of the breast found a lesion in the upper outer breast quadrant, which was consistent with a national cohort study that found the upper outer quadrant to be a common location for women who received mantle radiation for Hodgkin lymphoma [10]. Considering her predominantly negative genetic testing and that the patient confirmed she received historical mantle radiation that irradiated the site of her breast cancer, the diagnosis of secondary cancer was determined and thoroughly discussed with the patient. Following PET-CT and mediastinoscopy with a biopsy, the left breast was found to have Stage IV high-grade infiltrating ductal carcinoma, 2.7 cm, that is ER/PR-, HER2+ with mediastinal metastatic lymphadenopathy. She also had high-grade triple-negative Stage I right breast infiltrating ductal carcinoma. The plan is to attempt to cure her disease with neoadjuvant chemotherapy and immunotherapy with subsequent left axillary lymph node dissection, left mastectomy, and adjuvant immunotherapy.

## Conclusions

Mantle radiation has been found to cause secondary cancers. Therefore, it should be avoided if at all possible, and more modern radiation techniques with reduced radiation volume and intensity should be pursued to manage Hodgkin lymphoma. A thorough past medical history is imperative to identify risk factors that indicate the patient may benefit from earlier breast cancer screening and supplemental screening modalities to improve their likelihood of survival. While there are mixed recommendations regarding whether patients should perform a self-breast exam in the general population, for Hodgkin lymphoma survivors who received mantle radiation, perhaps self-breast exams should be considered. Patients should be encouraged to see their physician for an annual breast exam if they do not have access to mammography. Perhaps they should also consider annual breast exams if, for some reason, they are not a candidate for or refuse supplemental radiologic screening methods, as screening mammography has a 10%-20% false negative rate.

Clearly, there is insufficient evidence to establish a standard breast cancer screening regimen for Hodgkin lymphoma survivors who received mantle radiation from the 1960s to the 1990s in the United States. Future studies should evaluate breast cancer screening options for patients who received mantle radiation and consider self-breast exams. Early counseling and discussion with this patient population should emphasize that they have an above-average risk of developing breast cancer, and the initiation, frequency, and modality of screening should be developed through shared decision-making while respecting their autonomy.
